# Immunomodulation of periodontitis with SPMs

**DOI:** 10.3389/froh.2023.1288722

**Published:** 2023-10-20

**Authors:** Vaibhav Sahni, Thomas E. Van Dyke

**Affiliations:** ^1^ Immunology and Infectious Disease, The Forsyth Institute, Cambridge, MA, United States; ^2^ Faculty of Medicine, Harvard University, Boston, MA, United States

**Keywords:** inflammation, resolution of inflammation, periodontal disease, SPM, resolvins, lipoxins, protectins and maresins, oral health

## Abstract

Inflammation is a critical component in the pathophysiology of numerous disease processes, with most therapeutic modalities focusing on its inhibition in order to achieve treatment outcomes. The resolution of inflammation is a separate, distinct pathway that entails the reversal of the inflammatory process to a state of homoeostasis rather than selective inhibition of specific components of the inflammatory cascade. The discovery of specialized pro-resolving mediators (SPMs) resulted in a paradigm shift in our understanding of disease etiopathology. Periodontal disease, traditionally considered as one of microbial etiology, is now understood to be an inflammation-driven process associated with dysbiosis of the oral microbiome that may be modulated with SPMs to achieve therapeutic benefit.

## Introduction

### Historical perspective on inflammation

The medical principles of inflammation have been known since ancient times. Wounds and infections have always occupied a central space in the practice of medicine. Cornelius Celsus, a Roman physician is credited with being the first to explicitly define the clinical presentation of inflammation in 1 AD. He described four cardinal signs of inflammation: *rubor* (redness) and *tumor* (swelling) with *calor* (heat) and *dolor* (pain) while describing treatment procedures for chest pain in his treatise, *De medicina* (1). Along with other vascular changes specific to an acute inflammatory response, Waller and Cohnheim discovered the emigration of leukocytes from blood vessels, thereby establishing the physiological basis of the cardinal signs originally described by Celsus (1). Cohnheim further observed plasma leakage, vasodilatation and leukocyte emigration under a microscope. *Cellular pathologie*, authored by Rudolph Virchow, saw the addition of the disturbance of function (*function laesa*) as the fifth cardinal sign of inflammation (1). Virchow primarily contributed to the study of inflammation by establishing a cellular basis for the phenomenon, which was a paradigm shift from the more traditional view of humor imbalance. As such, *function laesa* is the only universal sign of inflammation, characterizing all its forms, while the original four were confined to be descriptive of only the acute phase.

1892 saw another milestone in the understanding of the inflammatory process when Elie Metchnikoff first described the process of phagocytosis and the cellular immunity theory. Metchnikoff stressed the favorable aspects of inflammation in his description of the role played by microphages (neutrophils) and macrophages in maintaining homoeostasis and host defense. Subsequently, Von Behring and Kitasato discovered diphtheria serum therapy, which led to the work of Ehrlich in developing the humoral immunity theory (1). Further development of the humoral immunity theory was provided by Bordet who discovered complement as an essential determinant of the immune response. Late in the 19th century, Pasteur and Koch established the germ theory of disease, which credited microbial insult as a major factor in the induction of an acute host inflammatory response.

### Overview of inflammation

Inflammation may best be described as the innate response of an organism to an insult by noxious stimulants such as injury or infection (2). An acute inflammatory response induced by noxious stimuli at its most basic level in mammals involves accumulation of leukocytes and plasma (blood products) to the site of insult (2, 3). In the context of this review of inflammation in periodontal diseases, we focus on inflammation induced by microbial challenge.

The innate inflammatory response is triggered by innate immune receptors called pattern recognition receptors, such as NOD-like (NLRs) and Toll-like receptors (TLRs). The microbial challenge is initially recognized and mediated at a local level by mast cells and tissue macrophages that elaborate a wider inflammatory response, including the production of various mediators such as cytokines, chemokines, eicosanoids and vasoactive amines (1). This results in the production of localized exudate at the site of insult. The exudate is due to the leakage of otherwise vascular-confined elements such as leukocytes and plasma proteins, which accumulate in extravascular sites via postcapillary venules. The initial stimulus leads to an activation of the vascular endothelium, which selectively confines erythrocytes, while allowing neutrophils to exit the vascular compartment. This preferential selectivity is the result of an interaction of leukocyte integrins and selectins with receptors present on the vascular endothelial cells (4). Neutrophil chemotaxis to the site of infection is followed by their activation either directly via pathogen contact or indirectly through cytokine action. The neutrophils attempt to eliminate noxious agents, a process characterized by the production of reactive nitrogen and oxygen species (5) and lysosomal granule release. At this stage, collateral damage to healthy host tissue may occur since the neutrophil degranulation response is not confined (6). An acute inflammatory response is deemed successful if it results in the elimination of noxious agents as well as inflammatory cells with a return of tissue homeostasis.

Apart from pathogens, foreign bodies and autoimmune responses may also be a cause for the development of an inflammatory response. Unsuccessful attempts to eliminate foreign bodies or pathogens results in granuloma formation, which is basically an attempt by macrophages to “wall-off” the noxious agent to protect the host (2, 3).

## Natural resolution of inflammation and specialized pro-resolving mediators

The acute inflammatory response may be divided into activation and resolution phases. Over time, evidence has accrued to suggest that the latter is not merely a passive process of chemoattractant and chemokine dilution which “fizzles out” after a while, but an active, programmed host response that “switches on” as the inflammatory lesion matures (7) that is mediated by a new class of eicosanoids collectively termed Specialized Proresolving Mediators (SPMs) (8–14). The discovery of this family of compounds introduced a fresh understanding of the inflammatory process, its development and indeed its resolution. Naturally, this led to a re-examination of previously established concepts surrounding disease etiology and treatment approaches. Building on previous knowledge, it is understood that the *in vivo* production of a mediator needs to be adequate to incite bioactions (11). The SPMs include resolvins, maresins, protectins and lipoxins that are derived from dietary Omega-3 polyunsaturated fatty acids (n-3 PUFA) or in the case of lipoxins, from endogenous arachidonic acid from cell membranes (15). Hence the concept of “good and laudable pus” which acknowledged the fact that pus contained the means to produce the resolution of inflammation. This family of compounds play an essential role in host defense by virtue of initiating the trafficking of leukocytes (16), a process known to be integral to the inflammatory response.

Upon challenge by a noxious stimulus, changes in blood flow, influx of neutrophils and edema are stimulated by prostaglandins and leukotrienes (17, 18). These processes manifest at a macro-level as the cardinal signs of inflammation. As the host inflammatory response builds up to this challenge, the pro-resolving mediators, maresins, protectins, resolvins and lipoxins along with their respective aspirin triggered (AT) variants undergo synthesis during the active phase of resolution (19, 20). It is imperative to recognize the synergy between the upregulation of pro-inflammatory elements of the response and the development of the resolution response itself.

The acute phase of inflammation may involve a microbial stimulus or one that is elicited because of the host's immune response to other forms of injury or insult. This is followed by local PUFA release from phospholipids found in cell membranes or their delivery to inflammatory sites by means of edema which would further result in the conversion of these PUFAs to specialized mediators (21).

**Figure 1:**
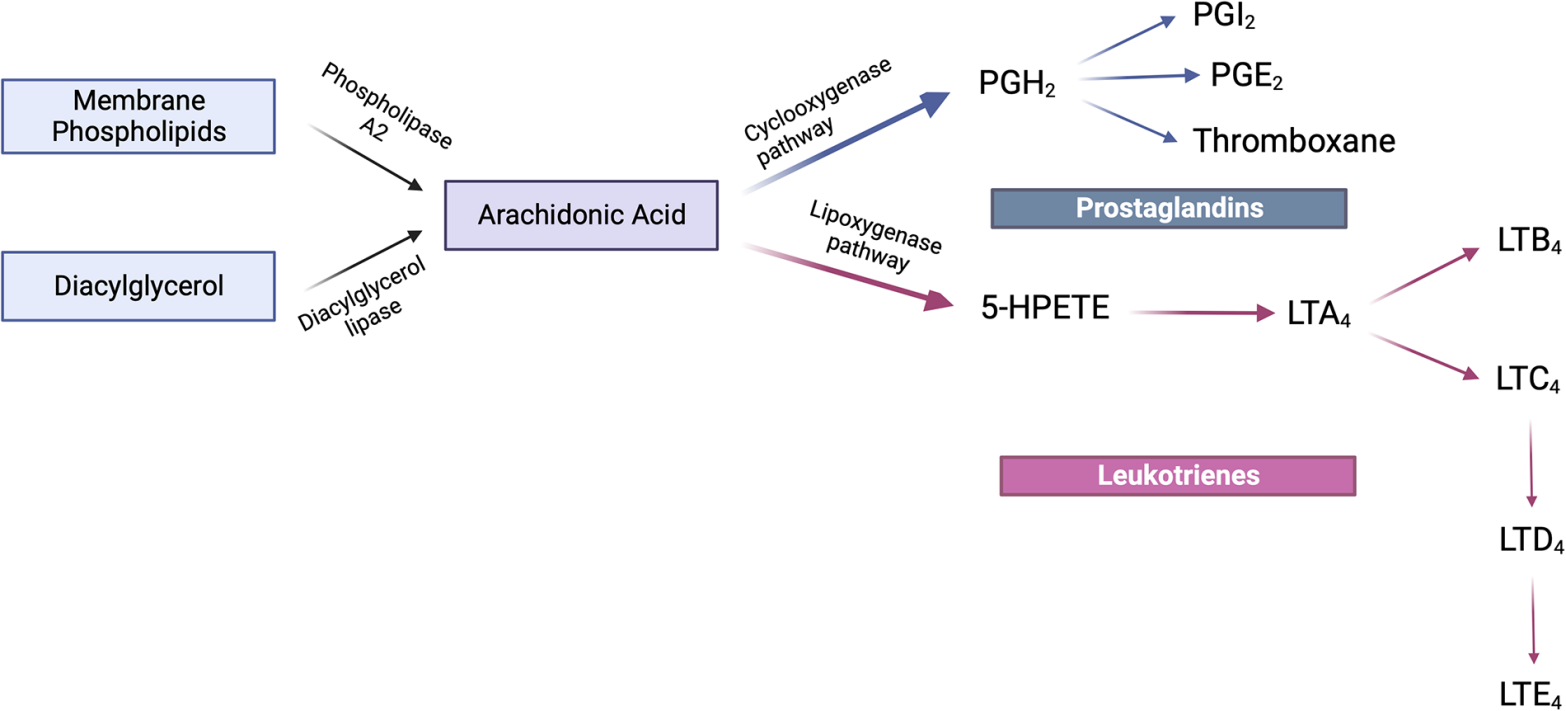
Arachidonic acid metabolism. Arachidonic acid is synthesized either from the action of phospholipase A_2_ upon membrane phospholipids or the diacylglycerol lipase-mediated conversion of diacylglycerol. The fate of this product is further determined by either the cyclooxygenase or lipoxygenase pathways. The cyclooxygenase-mediated pathway converts arachidonic acid to PGH_2_ which further elaborates the prostaglandins PGI_2_, PGE_2_ and thromboxanes. The lipoxygenase pathway leads to the formation of the intermediate 5-HPETE which is subsequently converted to LTA_4_. It is from LTA_4_ that LTB_4_ and LTC_4_ are formed of which the latter is converted to LTD_4_ which in turn forms LTE_4_. This family of compounds is termed leukotrienes. Created with BioRender.com.

**Figure 2:**
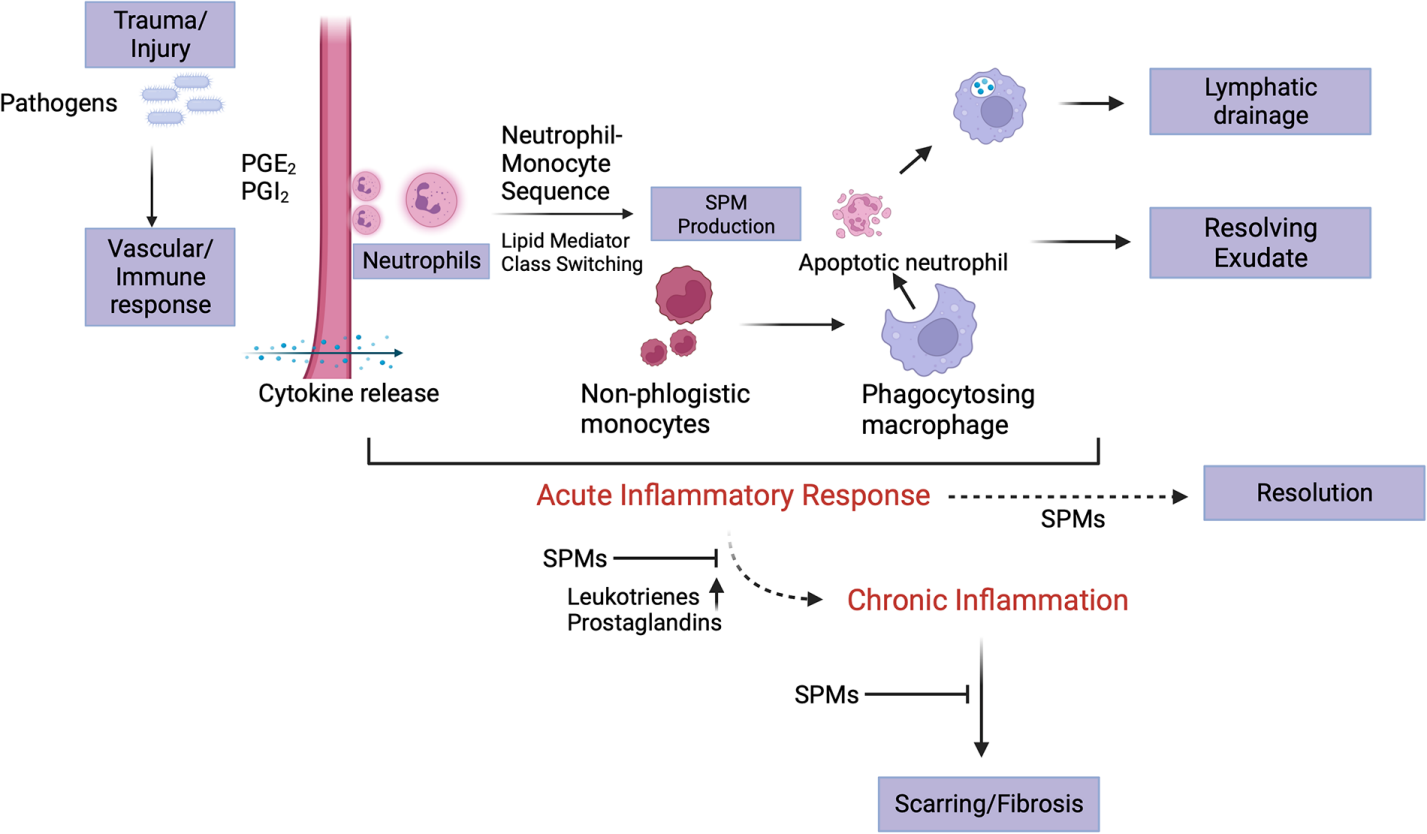
Acute inflammatory stage and its resolution. Eicosanoids play a crucial role in the initiation of inflammation subsequent to insult, which is manifest as the cardinal signs of inflammation. At the site of injury, trafficking of leukocytes forms part of the initial vascular response to the insult. Neutrophil migration is stimulated by LTB_4_, PGE_2_ and PGI_2_, the former being responsible for chemotaxis and adhesion while the latter two for vasodilation. An end to the acute phase of the inflammatory response is initiated by neutrophil-monocyte interaction that leads to a “class switch” in the lipid mediators (from eicosanoids to lipoxins). These lipoxins and then, resolvins signal non-phlogistic monocyte recruitment. The apoptotic neutrophils (“eat me” signals’) are subsequently cleared by the “big eater” macrophages in an SPM-mediated process termed efferocytosis. Once completed, this process paves the way for establishing homoeostasis. A failure in resolution may be marked by elevated levels of leukotrienes and prostaglandins, fibrosis and progression into the chronic phase. Created with BioRender.com.

Eicosanoids are produced from arachidonic acid within minutes of the initial insult (Figure 1). It is these leukotrienes and prostaglandins that signal neutrophils to arrive at the relevant site. Neutrophil availability is a function of LTD_4_ and LTC_4_ which impact vascular permeability, along with PGI_2_ and PGE_2_ which then affect blood flow (6, 22). As these products regulate neutrophil presence at the site of inflammation, the latter in turn tend to follow a transmigration gradient dictated by LTB_4_ (23). Once it is ensured that the right cells (neutrophils) are at the right place, neutrophil induction is mediated by these eicosanoids along with other immune system components such as the complement system, chemokines and cytokines (24, 25). It is relevant to understand these early events in the inflammatory phase since they are responsible for the subsequent lipid class-switching that occurs when the arachidonic acid metabolic system changes from leukotriene production to that of pro-resolving mediators such as lipoxins (26). If unencumbered, this process results in the natural resolution of inflammation wherein the acute phase proceeds to resolution (Figure 2).

**Figure 3:**
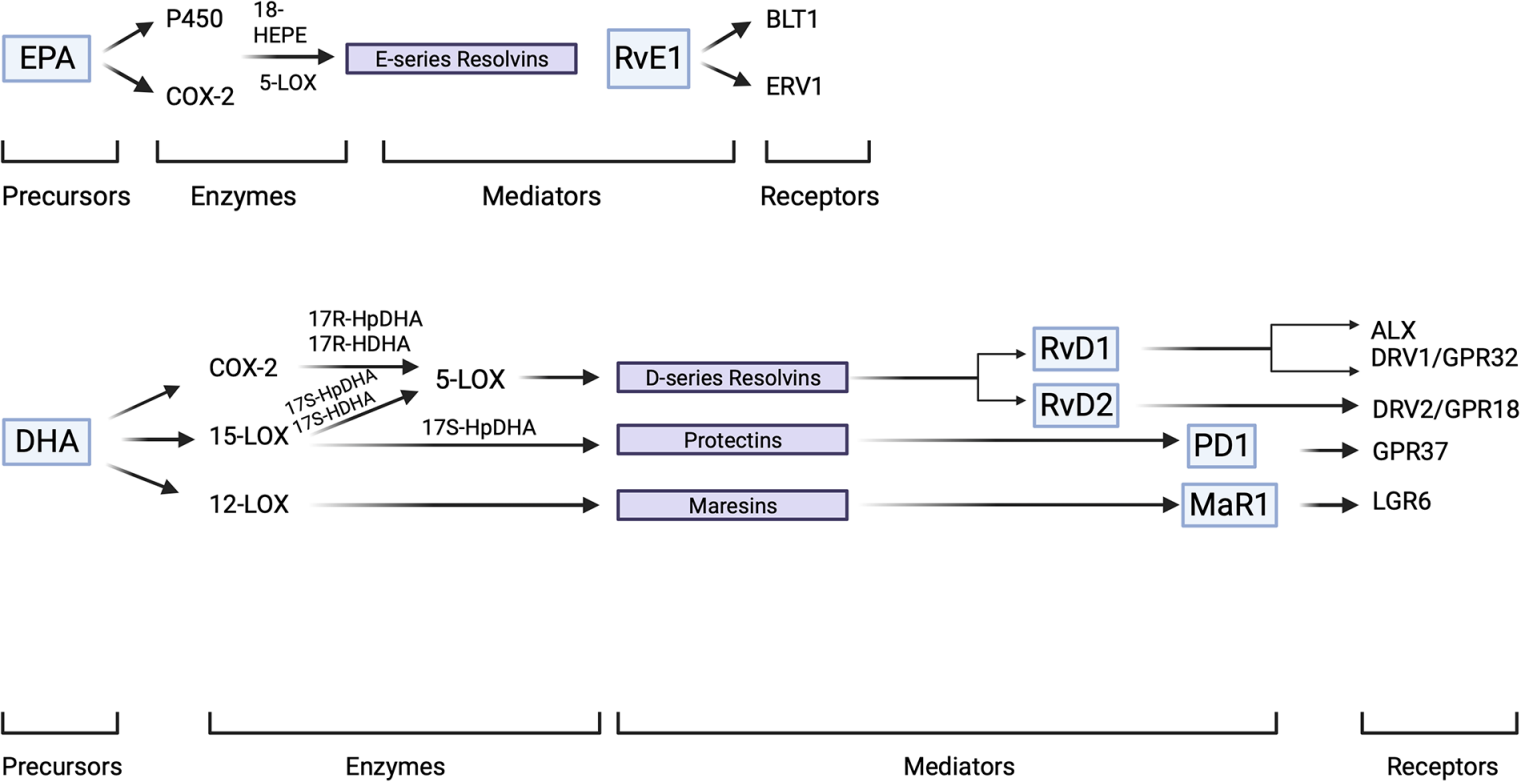
SPM overview. SPM precursors, EPA and DHA are enzymatically converted to SPMs which subsequently interact with their specific receptors to elaborate innate immune functions of a pro-resolving character. SPMs demonstrate stereoselectivity in their interaction with the corresponding GPCRs which result in the activation of intra-cellular signaling pathways. Created with BioRender.com.

The resolution response tends to get delayed in the event of an interruption with lipoxin production or of the availability of receptors for this family of compounds (27, 28). It is the acute inflammatory response that signals the development of the resolution phase. Essential fatty acids in the diet such as docosahexaenoic acid (DHA) and eicosapentaenoic acid (EPA) supplement the endogenous arachidonic acid-based system where these n-3 PUFA have been demonstrated to enzymatically (LOX-dependent) produce SPMs (Figure 3).

Thus, the production of these stereoselective products has been demonstrated via multiple pathways, which can be broadly categorized as intrinsic and extrinsic. The former includes SPM synthesis within the host in the absence of an external stimulant whereas the latter necessitates an external influence to the process. The intrinsic mechanisms for SPM production are varied based on enzyme mediators. One of these involves native enzymes, such as 12-LOX (platelet-derived) and 5-LOX (leukocyte-derived or 15-LOX (eosinophil-, monocyte- or epithelial cell-derived) and 5-LOX (leukocyte-derived) to produce lipoxins (21, 29, 30).

The extrinsic mechanism involves the modification of native enzymes by other compounds, such as aspirin. Even though aspirin inhibits the production of prostaglandins, it does so through a unique mechanism that involves modification of COX2 to produce a new active enzyme; not merely blocking the activity of COX2. It does this through acetylation of COX2 yielding a product that is an active 15R-lipoxygenase (31). When arachidonic acid or n-3 PUFA interact with this new lipoxygenase followed by 5-LO actions, the product is a longer acting epimer of lipoxins called aspirin triggered or epi-lipoxins. Statins have a similar property wherein they nitrosylate COX2 yielding a 15R-LO product (31). Intrinsically, cytochrome P450 enzyme complex may also produce 15R-HETE in the absence of a stimulus from aspirin (30–32). SPMs are present in exudates. Resolving exudates are also comprised of n-3 fatty acid derived pro-resolving mediators, including protectins, resolvins and maresins.

Resolvins of the E- and D-series are derived enzymatically from EPA and DHA, respectively, via intrinsic mechanisms whereas, COX-2 and LOX activities acetylated by aspirin also lead to generation of aspirin triggered epimers in a fashion similar to 15-epi-lipoxins (10).

The autacoids produced from EPA via these enzymatic processes are termed E-series resolvins (RvE). Similarly, the derivatives of DHA were termed D-series resolvins (RvD). Another family of chemically active products derived from DHA and characterized by the presence of a conjugated triene double-bond are named protectins. The nomenclature of resolvins was adopted to signify their potent immunoregulatory and anti-inflammatory actions (10). Protectins or neuroprotectins (protectins derived from neural tissues) were named to signify the protective character of 10,17-docosatriene and its anti-inflammatory nature (11, 33). EPA derived compounds such as 18R-HEPA and 18R-HEPE have also been demonstrated to be present in resolving exudates (8). Transcellular synthesis of RvE1 in the vasculature, in the presence of aspirin, resulted in the formation of 18R-HEPE from EPA mediated by endothelial cell derived COX2 and further, leukocyte derived 5-LOX mediated RvE1 formation utilizing 18R-HEPE as the substrate (8, 34). The two major resolvin series derived from DHA include the RvD1-6 and their aspirin triggered isomers (AT-RvD1-6) (35).

17S-hydroperoxy-DHA derived from DHA through an enzymatic reaction mediated by 15-LOX and later by 5-LOX leads to the formation of the D-series of resolvins. In the case of their aspirin triggered epimers, 17R-HpDHA is derived from DHA through a mechanism mediated by COX-2 acetylated by aspirin also subsequently acts as a 5-LOX substrate for an eventual epimeric variant transformation.

Not just resolvins and protectins but maresins are also groups of additional compounds derived from DHA that have been observed in resolving exudates and exhibit pro-resolving and protective activities (11, 12). This is evidenced by the fact that 15-LOX mediated formation of protectin D1 from 17S-HpDHA and 12-LOX mediated maresin 1 (MaR1) formation from 14S-HpDHA have been observed at sites of inflammation (31, 36).

It is essential to understand how significantly the process of resolution differs from that of inhibition. Traditional therapeutic methodologies have focused on anti-inflammatory therapies, which are based on the concept of inhibiting the proinflammatory process. However, in doing so, they also cut-off the signals necessary to initiate and sustain the process of resolution. This is a critical caveat since the process of resolution is central to the favorable outcome of the inflammation cascade, since it reverses the inflammatory process and signals a return to homoeostasis and restoration of cellular structure. While anti-inflammatory strategies act to block enzymes or antagonize relevant receptors, the SPMs exert their activity in an agonistic manner through their interaction with their corresponding receptors. A G-protein coupled receptor, FPR2/ALX binds 15-epi-LXA_4_ and LXA_4_ with significant affinity. Other receptors for SPMs have also been identified such as GPR32 for LXA_4_ chemR23/CMKLR1/ERV1, BLT1 for RvE1, GPR32/DRV1, ALX/FPR2 for RvD1, GPR18/DRV2 for RvD2, GPR32 for RvD3, GPR37 for PD1 and LGR6 for MaR1 (37–39).

There appears to be an overlap amongst some of the receptors in terms of binding compounds from the SPM family. A notable finding has been that of RvD1 exhibiting equipotent activity to LXA_4_ upon binding with ALX (37). Further, AT-RvD1, RvD3 and RvD1 all demonstrated high affinity binding to GPR32 (37). The understanding of these pathways is significant since disruption leads to impaired resolution that, in turn, leads an acute inflammatory lesion to progress into a chronic state (27, 40). This disruption in the resolution response may occur as a result of inadequacies in enzyme synthesis, receptor expression, essential PUFA deficiency or intra-cellular signaling. Even though the families of these immunoresolvents are structurally distinct, it is their common functional role in the resolution of inflammation that unites them as SPMs. Studies from experimental models have demonstrated these limit damage to tissues, promote healing, control the inflammatory response, decrease the interval to resolution and alleviate pain. These compounds do not act alone but are components of a larger resolution response of the body, which includes cytokines, annexin A1 protein, carbon monoxide and microRNAs and cyclin dependent kinase inhibitors (9, 41–44).

It was over a century ago that Metchnikoff noted that macrophagic clearance of neutrophils tended to resolve inflammation (16). As subsequent research identified the initiators of leukocyte recruitment to sites of insult, the thought process which evolved was to either block these signals or remove the initiators of inflammation in order to prevent inflammation. It was believed that the chemoattractants so involved, gradually undergo dilution and dissipation in a passive manner to end the inflammatory phase. It appears that the fundamental processes surrounding the resolution of inflammation, such as removal of apoptotic PMNs (polymorphonuclear neutrophils) and cessation of tissue-entry of PMNs are impacted by SPMs. This has been demonstrated in studies which have observed in resolving exudates, that cellular interactions expound signals to impair the influx of neutrophils and upregulate macrophagic clearance of these as well, both considered key processes for the resolution of inflammation.

It has been demonstrated that prostaglandins participate in the initial stages of inflammation impacting the production of pro-resolving mediators by stimulating mRNA translation for the responsible enzymes (26). This essentially is a “class-switching” of the lipid mediators to go from ones which are pro-inflammatory to ones which are pro-resolving in action. Temporal analyses of exudates point towards the biosynthesis of lipoxins subsequent to an early appearance of prostaglandins and leukotrienes. The appearance of lipoxins has been correlated with the resolution phase of inflammation (45). Macrophages of the M1-like and M2-like type have been observed to exhibit different responses to stimulation by pathogens and pharmacological inhibitors. The difference lies in the distinction between the lipid mediator profiles that are produced, which further characterizes the pro-resolving and inflammatory cell phenotypes. PGE_2_ and LTB_4_ are produced by M1-like macrophages and a simultaneous stimulation of the M2-like phenotype as a result of a challenge from *S. aureus* and *E. coli* (11, 46). From this, one may appreciate the significance of the lipid mediator class switch, which appears to impact the inflammatory cascade at numerous levels. It has also been observed that an inhibitor of microsomal prostaglandin E_2_ synthase-1 and 5-LOX selectively downregulates PG and LT production in the M1-like phenotype but enhances maresin and resolvin production in the M2-like variant (47).

Further credence to the extrinsic mechanism of SPM production can be evidenced by the observation that Resolvin E2, another bioactive member was identified when the common E-series precursor 18-HEPE was subjected to human recombinant 5-LOX (48). In general, SPMs being agonists have the capacity to signal important events in the resolution phase such as stimulating the clearance of apoptotic cells by macrophages and downregulating neutrophil infiltration (15). The pro-resolution and anti-inflammatory processes are not alike. The former involves agonists (SPMs) to halt the influx of neutrophils and stimulate non-phlogistic macrophage responses, these two cardinal features of resolution lead to a restoration of homoeostasis (49).

Every SPM has characteristic proresolving activity that is of significant importance to the resolution of inflammation. These include stopping or limiting neutrophil influx, cytokine/chemokine counter regulation, pain reduction and stimulation of nonphlogistic macrophage activity (50). SPMs act as agonists binding to receptors on multiple targets (neutrophils, macrophages, stromal cells) individually in order to bring about resolution. SPM production in humans is temporal with SPMs acting selectively by signaling resolution to control inflammation without being immunosuppressive (50, 51). In periodontal, kidney, eye and lung diseases, their actions have been demonstrated to be organ protective (50, 51).

## SPMs, diurnal variation, and hormonal influence

SPM production demonstrates a diurnal pattern of regulation. A central mechanism requisite for the maintenance of resolution at the tissue level has been elucidated to involve the biosynthesis of pro-resolving mediators by acetylcholine (Ach) release. Ach levels have been demonstrated to be high in the early hours of the day in healthy volunteers. Blood from these volunteers when incubated with Ach demonstrated an upregulation of RvD_n_-_3 DPA_ (52). Reduced plasma levels of RvD_n_-_3 DPA_ in the early morning hours, essentially a loss of the diurnal regulation mechanism, has been postulated to contribute to the onset and propagation of cardiovascular disease (CVD) (52).

Uncovering the dysregulated circadian rhythmic production of pro-resolving mediators in various disease processes may aid in developing therapeutic strategies, especially in light of studies that have demonstrated the circadian rhythm regulated production of IL-6, TNF-alpha and Ly6c^hi^ monocytes (53, 54).

The role of hormonal regulation of SPMs has been demonstrated in a selective and rapid upregulation of SPMs characterized by a typical profile including RvD1 and RvD2 observed in mice administered parathyroid hormone (PTH), which triggers a pro-resolving circuit in the backdrop of bone remodeling (55).

Androgen DHT and estrogen have been demonstrated to downregulate the action of the pro-inflammatory LTB_4_. Indeed, a sex-based difference has been observed in the reaction of cultured conjunctival goblet cells to LXA_4_, with human male cells demonstrating a greater Ca^2+^ response (56). Similarly, in human tear samples, it was found that males demonstrated higher levels of RvD1 and RvD2 (57).

These findings suggest that future studies should further evaluate sex-based differences in SPM production and response also controlling for time of day.

## The role of SPMs in infectious diseases

The inflammatory process has time and again been demonstrated to be a key underlying factor in the pathophysiology of numerous infectious disease processes. Naturally, a family of compounds that act to resolve this inflammation would potentially be of immense benefit in not only understanding the disease but in therapeutics as well. Recently, scientific literature has discussed the role SPMs may play in host modulation for several infectious diseases, which provides the scope for exploring a potentially novel therapeutic modality.

Inflammation has a central role to play in pneumonia, a highly prevalent disease of concern both at the community- and hospital acquired-levels. Typically, pneumonia elicits an acute response which is self-limiting in nature. In certain cases, this may progress into acute respiratory distress syndrome (ARDS) which is characterized by respiratory failure and hypoxemia (58). In cases of pneumonia induced by *Escherichia coli*, Lipoxin A_4_ (LXA_4_) has been observed to stimulate BCL-2-associated death promoter (BAD) phosphorylation and decrease the expression of myeloid cell leukemia sequence 1 (MCL1), which causes neutrophil apoptosis (58). RvE1 has also been observed to stimulate neutrophil apoptosis but via a different mechanism of activating caspases (58). In both the above situations, the severity associated with lung inflammation in its acute phase is blunted as a result of neutrophil death. Additionally, in a mouse model of pneumonia induced by *E. coli*, RvE1 has been demonstrated to decrease local pro-inflammatory cytokine production and promote bacterial clearance (59). It was shown that the actions of SPMs aid in *E. coli* clearance and the decreased levels of host inflammation, which enhanced survival (59). It is quite apparent that resolving the inflammatory process provided the added advantage of microbial clearance, which tends to act as the initiator in a number of disease processes.

Another infectious disease of bacterial etiology (but tick vector), Lyme disease, involves an inflammatory component that manifests after exposure to the initial etiological agent, which microbial in nature. Mouse models of Lyme disease have helped elucidate the systemic and local control of inflammation (60). In mice with an impaired SPM production mechanism, i.e., those deficient in 5-LOX, infection with *Borrelia burgdorferi* caused a similar pattern of arthritis development as it did in wild type counterparts, the key feature, however, was that the lack of resolvins and lipoxins impaired the ability of the host to resolve arthritis, giving rise to chronic disease, the inflammatory response of which persisted long after the causative agent was cleared (60).

Tuberculosis, in both its typical pulmonary and extra-pulmonary forms, is a disease of concern particularly in the developing world. In a pattern similar to other infectious diseases, it involves both a microbial and inflammatory component. The host response to *Mycobacterium tuberculosis* involves a balance between pro-inflammatory and pro-resolving mediators, which determines microbial clearance as well as pathogen induced inflammation (61). In a mouse model, it has been demonstrated that infection with *Mycobacterium tuberculosis* led to an increase in the levels of LTB_4_ (pro-inflammatory) and LXA_4_ (pro-resolving) with high levels of the latter being maintained during the chronic phase of the infection (61). Further, 5-LOX deficient animals have demonstrated increased survival with *M. tuberculosis* infections (61). This demonstrates the importance of the inflammatory response in determining disease outcomes. As was discussed previously, it is the inflammatory response which begets the generation of the resolution component. The *M. tuberculosis* strain has been related to lipoxin generation in the host which points towards SPMs effecting host modulation of the inflammatory response in tuberculosis infections (62). Either LXA_4_ or LTB_4_, if produced in excess, may result in a skewed response of the host to *M. tuberculosis* infection, which related to a dysregulation in the expression of tumor necrosis factor (TNF) (62).

Hence, both the pro-resolution and pro-inflammatory pathways seem to be important in determining host defense and outcomes of pathogen caused inflammation (63). It would appear that a therapeutic modality involving antibiotics to clear the pathogen along with SPMs to manage the immune response would be effective. This is further given credence by the finding that varying rates of infection were observed for human variants for the LTA4H and ALOX5 loci, both of which interfere with LXA_4_ and LTB_4_ production (64, 65).

Perhaps the most serious complication arising from a bacterial infection in its acute phase is that of sepsis. The host response in such cases tends to involve a rapidly progressing system-wide immune dysregulation which may result in shock (66). In a mouse cecal ligation puncture model, treatment of sepsis with LXA_4_ downregulated pro-inflammatory cytokine production and reduced gram-negative bacterial numbers, which resulted in improved survival (66, 67). RvD2, by means of downregulating the activity of nuclear factor-κB (NF-κB), regulates pro-inflammatory signals and thereby the overall inflammatory response at the systemic level (50, 59, 66, 67). As was demonstrated in a mouse model of sepsis, administration of RvD2 led to a significant decrease in cytokine production, particularly that of IL-10, IL-6 and interferon-α (IFN-α) (43). There was also reduced infiltration of leukocytes at the infection site (50). It is interesting to note that controlling the inflammatory response in such a manner brings about an overall reduction in bacterial counts both at the systemic and local levels along with an improvement in the overall survival of these animals (50).

Another consideration is one involving the timing of SPM administration. It was demonstrated in a mouse pneumosepsis model that treatment with LXA_4_ in the early stages limited the immune response since it reduced leukocyte infiltration. It was also noted that these mice had a deterioration in their survival rate along with impaired bacterial clearance (68). On the contrary, when LXA_4_ was utilized in the later stages of treatment, it demonstrated certain positive effects such as satisfactory infection clearance while blunting the protraction of the inflammatory response and thereby improving survival (69). By means of their activity affecting bacterial clearance and downregulation of the pathological inflammatory response, SPMs have been demonstrated to decrease antibiotic dosage requirements for clearing bacterial infections (67, 69). This finding assumes significance in the face of the growing global threat of antibiotic resistance.

SPMs also regulate the mechanisms by which certain viral pathogens interact with the host. In a comparison between influenza strains of varying virulence, it has been observed that the more virulent strains cause greater suppression of lipoxins and hence, an enhanced dissemination of the virus within the host (70). Protectin D1 levels have been observed to be downregulated by highly virulent influenza strains such as H5N1, which reinforces the inverse relationship between SPM activity and strain virulence (71). Apart from modifying the host immune response, protectin D1 and the isomeric protectin DX limit viral replication by scrambling the nuclear export mechanism (11, 45, 46). In fact, protectin D1 has been observed to exhibit better clinical outcomes in cases of influenza virus infections (72). Infected mice, treated with protectin D1 even as late as 48 h into the infection, reported an improvement in survival (72). This finding appears to be important when contemporary antiviral therapies seem to be declining in effectiveness (73). Infection with the respiratory syncytial virus (RSV), another respiratory pathogen of considerable relevance, is characterized by macrophage-driven bronchiolitis which is subsequently resolved by their alternatively activated counterparts (74). This may be explained by the fact that alternatively fated macrophages appear to have a relation to RSV mediated COX-2, RvE1 and LXA_4_ related protective action (74, 75).

Herpes Simplex Virus (HSV) infections tend to be particularly distressful and recurrent in their presentation with the potential to progress into states of morbidity. Ocular HSV infection presents a useful model to demonstrate control of the virus at the local level via a strong inflammatory response, the chronicity of which may have significant morbidity. In mice with ocular herpes, it was observed that treatment with topical RvE1 was associated with a reduced number of neutrophils, Th17 and Th1 cells in the cornea (50). The mechanisms for this were hypothesized to be multiple, including a downregulation of CD4^+^ T cells and neutrophil influx, increased anti-inflammatory mediator production and reduced production of pro-inflammatory mediators (76). One study in particular demonstrated that RvE1 may be utilized to regulate severity of lesions in immunopathologies of a viral origin (75). This essentially provides evidence of SPMs regulating the immune response initially elicited by microbial stimulus. The timely involvement of SPMs in the inflammatory cascade is crucial to the outcome of the inflammatory response.

Based on findings from a mouse model it has been suggested that lipoxin induction in response to stimulation by *Toxoplasma gondii* extract serves as a potent pathway for regulating the function of dendritic cells during the host innate immune response (77). Wild type mice exposed to *T. gondii* infection produced LXA_4_ during the early stages of the chronic infection, whereas mice deficient in 5-Lipooxygenase [5-LO(−/−)] exhibited shorter survival during the same time frame characterized by encephalitis (78). The mortality of these mice was attributable to greater levels of IFN-gamma and IL-12 and was prevented altogether by treatment with a stable analogue of LXA4 (78), again evidencing the essential role played by SPMs in determining the outcome of inflammation. Lipoxins have an alleged protective role in other parasitic infections such as those involving *Plasmodium* species, *Angiostrongylus costaricensis* and *Trypanosoma cruzi* (79–81).

### Specialized proresolving mediators (SPMs) and the return to tissue homeostasis

Class-switching of lipid mediators from pro-inflammatory to anti-inflammatory or pro-resolution is imperative for initiation of the resolution phase of acute inflammation. This class-switch from prostaglandins and leukotrienes to pro-resolution elements leads to an inhibition of neutrophil recruitment in favor of monocytes, which remove cellular debris and set the stage for tissue remodeling (82). If the acute phase fails to enter the resolution phase, due to failure of production of adequate proresolving mediators or failure to eliminate the noxious stimulus, the inflammatory response becomes chronic and is characterized by persistence of inflammatory macrophages and T cells, which involves tertiary lymphoid tissue and granuloma formation (3, 51).

The inflammatory process and production of relevant mediators is temporal. Thus, along with the production of mediators to promote proinflammatory processes, there is an inherent feature of local termination of inflammation. These resolution pathways are activated by the proinflammatory pathways. Prostaglandins activate transcription of enzymes (12- and 15-lipoxygenases) that are required to produce lipoxins, resolvins, protectins and maresins that promote resolution of inflammation through specific receptors. There are multiple receptors for the various SPMs that have been reported. This interaction has been described as the *alpha* signaling the *omega* or the beginning signaling the end to indicate that the class-switch that occurs to produce molecules responsible for the resolution phase is initiated by the pro-inflammatory response of the initial stages.

The characterization of these compounds has changed the understanding of resolution of inflammation from the earlier belief that resolution was a passive process mediated by decay of proinflammatory mediators to an understanding that SPMs actively promote a resolution response. These new lipid mediator pathways open the door for novel therapeutics, since these lipids can be produced by complete organic synthesis, are small molecules (below 500 Da) and can be manufactured utilizing contemporary pharmaceutical methods. Both animal and human studies have revealed SPMs to be of benefit in the resolution of the inflammatory response, which is characteristic of several infectious diseases as well as periodontal disease. It is already known that such disease processes involve a microbial etiology that initiates the lesion in a susceptible host. In this review, we will explore the relationship that resolution of inflammation has with dysbiosis, for it is an interplay of these processes that eventually leads to a favorable therapeutic outcome.

## SPMs in periodontal disease

### Inflammation and periodontal disease

The etiology of periodontal disease is multifactorial, being both host-specific and polymicrobial. While microbial challenge may be the initiator of the disease process, the pathogenesis is inflammatory in nature (83, 84). The American Academy of Periodontology considers periodontitis as an inflammatory disease with bacterial initiation (9). Even though this definition represents a paradigm shift in the understanding of the pathogenesis of periodontal disease, there remains a lack of clarity regarding the relationship shared by the disease itself and the inflammatory response in that, it remains unclear whether the immune response preceded the dysbiosis or vice versa (85). There is evidence in literature to suggest that host immune responses tend to shape microbial communities in biofilms on mucosal surfaces (67). However, the extent to *which* bacterial biofilms play a role in triggering human disease is still under active investigation and debate (86–88).

Mucosal inflammatory lesions have been evidenced to occur as a result of the presence of biofilms at numerous locations in both animals and humans (86, 89). At the same time, in entities such as chronic rhinosinusitis and inflammatory bowel disease, even though biofilms have been detected on mucosal surfaces, their formation may be attributable to a deranged immune response upon stimulus from antigens of a non-microbial origin (90, 91). Hence, it would appear rational to argue that an immune response to non-microbial antigens may produce conducive environments for biofilm development. It is already known that microbes within biofilms behave differently when compared to their planktonic counterparts. Cells within biofilms tend to possess greater resistance to host defense mechanisms on account of an increase in biomass which impedes phagocytosis and extra-cellular polysaccharides that act as a physical barrier to antibodies, immune and complement systems (86). The biofilm need not even be extremely pathogenic, but an ineffective and hence, “frustrated” host immune response may end up causing collateral damage to surrounding tissues (86).

The primary etiologic agent of periodontitis is recognized as an insult originating from a pathogenic biofilm, but a specific bacterium or group of bacteria is not established as causing periodontitis (92, 93). Cross-sectional studies have implicated the “red-complex” bacteria, i.e., *Porphyromonas gingivalis (P.g.)*, *Treponema denticola (T.d.)* and *Tannerella forsythia (T.f.)* to be associated with the presence of periodontitis, the causality for this is yet to be established in longitudinal studies (72). It is however, known that in susceptible hosts, the immune response to biofilm leads to a loss of tissue as part of the pathogenesis of periodontal disease (92).

Traditionally, therapeutics have revolved around the biofilm spectrum of disease, generally aimed at removing the aetiologic factor. It has been acknowledged that the bacteria being dealt with are present in large numbers and are spread across a multitude of sites (94). Moreover, these exist as biofilm-based communities which aid in their defense against antimicrobials and host response (94). Additionally, the microorganisms possess a formidable potential for multiplication and attachment to both like and unlike surfaces (94). Naturally, these factors hinder traditional therapeutics involving biofilm removal strategies, an imperfect treatment modality. At best, traditional periodontal therapies provide some benefit impeding or ceasing the progress of periodontal disease (94). These methods are, however, not always successful and may require adjunctive or alternative therapeutic measures (94). Antimicrobial therapies for the treatment of periodontal disease may include physical debridement, ones aimed at killing or altering the metabolism of the microorganisms in question or ones which alter the environment in which these microorganisms exist (94). Treatment modalities based on mechanical methods require accessibility to the biofilms to be rendered effectively. Fortunately, dental biofilms are quite accessible and are amenable to attempts at mechanical debridement. Given their ability to resist antimicrobial agents and host response, it would be logical to initially attempt a physical means of control for these microorganisms.

No doubt, mechanical therapy is able to remove a vast majority of microorganisms, however, the enormous multiplicative potential of bacteria allows these numbers to return to roughly baseline numbers within 3 months of therapy (94). There is also evidence in literature to suggest a return to baseline total counts within 4–8 days of therapy (95, 96). Since there appears to be no singularly isolated microorganism identified as the etiological agent for periodontal disease and in light of the fact that traditional therapeutic measures fall short of providing satisfactory results, let alone a “cure”, it would be logical to examine the relationship between inflammation and dysbiosis.

Periodontitis entails a significant increase in pro-inflammatory mediators, particularly prostaglandin E_2_ (PGE_2_) and leukotriene B_4_ (LTB_4_), which are generally considered lipid mediators related to early stages of the host response (97). Gingival Crevicular Fluid (GCF) has been reported to have increased exudative flow rates in states of active inflammation and has also been utilized as an analyte of interest being representative of the local and systemic inflammatory response. Increased GCF levels of pro-inflammatory markers such as PGE_2_, in periodontal disease can be correlated with disease activity and severity along with being potentially utilized to predict future disease progression (97). Another example of the relationship between the immune and microbial spectrum of disease pathophysiology is provided by *in vitro* studies that have demonstrated an increase in COX-2 activity as a result of stimulation from periodontopathogens (98).

As has already been discussed, neutrophils act as the “first responders” to sites of inflammation and form a crucial component in determining the progress of the lesion. Initially, it was demonstrated that neutrophils isolated from patients with periodontal disease, particularly those with the localized aggressive variant (as classified at the time), produced greater levels of LXA_4_ when compared to controls (98). Subsequently, *in vivo* priming of neutrophils and increased LXA_4_ levels were demonstrated in patients with asthma, a non-infectious inflammatory condition (99). Similar outcomes were demonstrated *in vitro* via cytokine induction (100). From such data it was argued that lesions of chronic periodontal disease featured a hyperactive neutrophil population which resulted in activating lipoxin production pathways. It is interesting to note that either due to dysfunctional receptor binding or insufficient quantities of lipoxins, the inflammatory phase was not resolved if a natural production of resolvins was relied upon (101). That being said, adequate quantities of lipoxins were protective against tissue damage in periodontal disease induced by bacteria (101). This was evidenced in a rabbit model which revealed that rabbits overexpressing 15-LO had decreased recruitment of neutrophils and in turn, lower levels of potentially tissue harming enzymes being released (101).

Further evidence as to the role of lipoxins in protecting against damage caused by periodontal disease is provided from findings in patients of localized aggressive periodontitis in whom LO activity appeared to be somewhat aberrant in nature (98, 102). The platelets of such patients featured an increase in the surface expression of P-selectin while the monocytes and neutrophils expressed an upregulation in CD18 expression (102). This was correlated with greater platelet-monocyte and platelet-neutrophils aggregations in the whole blood of these patients (102). These findings point towards the role of SPMs in protecting the host against bacterial-induced periodontal disease.

The pathogenesis of periodontitis may be regarded as being a result of the robust and protracted host immune response to a microbial challenge presented by organisms such as *P. gingivalis* and *Actinobacillus* spp. (103). The control of periodontal disease also attenuates the immune response to disease at a systemic level, which is characterized by blunted systemic inflammation as represented by reduced CRP levels and reduced interactions between neutrophils and platelets (103–105). It has been demonstrated in a rabbit model of ligature and *P. gingivalis* induced periodontal disease, that topical application of RvE1 to control inflammation leads to a reversal of changes in the biofilm associated with disease and essentially constituted a resolution of dysbiosis with *P. gingivalis* elimination (103). SPM usage does not have an identifiable side effect profile and long term, or recurrent usage does not increase susceptibility to infection. Since SPMs function to resolve the acute phase of inflammation, their utilization as therapeutic agents in the initial or early stages of periodontal disease provides a benefit (104).

Topical treatment with RvE1 has been demonstrated to sharply diminish the inflammation related to increased activation of neutrophils in the periodontium, elucidating a role for this class of lipid mediators to modify *in vivo*, local inflammatory processes presenting as a result of microbial challenge (106). RvE1 was also demonstrated to directly inhibit osteoclast induced resorption of bone, which was expressed as the ability of RvE1 treatment to prevent the appearance of TRAP+ cells in the bone (106). These findings demonstrate a more favorable outcome of inflammation when pro-resolution treatment strategies were employed instead of the traditional anti-inflammation regime.

## Inflammation and dysbiosis

Inflammation was initially described as possessing certain cardinal signs. These may be considered the result of endogenous and exogenous chemical mediators released as a result of insult. Microbial peptides may act as exogenous mediators which serve as chemoattractants to signal neutrophil infiltration at the site of insult, where these may act by phagocytosing cell debris and microorganisms (107). Phagosomes within neutrophils fuse with lysosomal granules to mature into phagolysosomes that contain enzymes and reactive oxygen species to process the entrapped debris and microorganisms (107). At this stage, the initial inflammatory response can be viewed as one which is protective in nature and its timely resolution would ensure that it is self-limiting as well (107).

A failure of timely resolution of inflammation due to any number of factors would generally mean that the acute inflammatory episode would progress into a more protracted phase of chronic inflammation (108). This is likely to happen in situations where biofilms are involved wherein the microbial community is able to mount a sufficient defense against the host response leading to collateral damage (108). A great deal of theoretical knowledge surrounding the concept of dysbiosis in periodontal disease is based out of experience with research of the gut microbiome. With the complexity of biofilm structure and the potential multitude of host responses it may elicit at various levels, it would not be completely unfair to hypothesize that no one particular microorganism or “keystone” pathogen would be responsible for the initiation of disease. Indeed, there is no such evidence in literature to support such a finding (108). This adds further credence to the role of the inflammatory process in the pathogenesis of periodontal disease. Further, it is recognized that bacteria involved in disease initiation are generally commensal in nature and the so called periodontopathogens form a very minor portion of the biofilm in its early stages (109).

The ecological plaque hypothesis was the first to recognize that dysbiosis of the microbiome would be the result of a persistent and excessive inflammatory response along with pocket formation, which essentially alters the environment in which microorganisms grow by exerting selective pressure upon other specific microorganisms (110). The microbial community pertaining to the periodontium remains generally stable in a state of health while maintaining a dynamic equilibrium (108). Gingivitis may be considered as representative of homoeostasis in that it tends to remain a stable inflammatory lesion (108). It is a failure of timely resolution of inflammation that slides into a chronic phase leading to a loss of tissue (108). Bacteria associated with periodontal disease increase in significant numbers with the progress of the lesion (108). Similarly, a shift to healthy state also involves a change in the composition of the bacterial community (108, 111, 112).

While there is no doubt that polymicrobial insult causes gingivitis, whether the disease progresses is determined by the host response to this insult. This assumes particular importance when one considers the wealth of evidence implicating the host response as the factor responsible for driving tissue destruction (113, 114). Recent literature has suggested that the host immune response may dictate biofilm composition (103). Essentially, this implies that the inflammatory response in turn causes an alteration of the biofilm microenvironment and leads to specific selection of microorganisms. From this, one could infer that inflammation in the periodontal pocket would precede the overgrowth of certain periodontopathogens such as *P. gingivalis* and *Tannerella forsythia* at this site (115). Naturally, the initiating factor of disease falls into question. Microbial diversity appears to be stable in both health and severe forms of periodontal disease, whereas it is in shallow pockets that a greater diversity of microorganisms has been observed. A longitudinal study by Tanner et al. demonstrated that there appeared to be no particular microorganisms that preceded the occurrence of attachment loss. They found that gingival inflammation was the only reliable predictor of attachment loss (115).

Accumulating evidence of these concepts over the years has rendered the initial assumption of specific periodontopathogens causing disease as invalid. Coming back full circle to the ecological plaque hypothesis, one may view periodontal disease pathogenesis as the result of plaque accumulation which elicits an inflammatory response that in turn alters the microenvironment of the biofilm, specifically selecting for certain “pathogens” to overgrow, which further magnify the inflammatory response and enter a cyclical process of disease progression. Indeed, it has been demonstrated in a rabbit model that the progression and even onset of *P.g* induced periodontitis was prevented by Resolvin E1 (RvE1) by greater than 95% (106).

An overview of SPMs, their precursors, target cells and cell-specific activity is provided (Table 1).

**Table 1:** An overview of SPMs, their precursors, target cells and cell-specific activity.

SPMs	Precursors	Target cells	Cell specific actions
Lipoxin A4	Arachidonic Acid	Neutrophils	Inhibits degranulation, generation of superoxide anions, interactions with epithelial cells, trans-epithelial and endothelial migration, chemotaxis (116–121).
		Monocytes	Inhibits generation of peroxynitrite, decreases release of IL-8, stimulates adhesion and chemotaxis (122–124).
		Macrophages	Upregulates apoptotic neutrophil engulfment (125).
		Eosinophils	Inhibits chemotaxis, migration and generation of IL-5 and eotaxin (80, 126).
		NK cells	Upregulates apoptosis of granulocytes, inhibits cytotoxicity (127, 128)
		Dendritic cells	Inhibits production of IL-12 (129).
		ILC2	Inhibition of IL-13 release (127).
		Endothelial cells	Inhibition of cell migration induced via VEGF, prevents ROS generation, upregulates formation of prostacyclin (PKC-dependent) (130–132)
		Epithelial cells	Prevents release of IL-8 and IL-6, upregulates proliferation subsequent to acid insult (119).
		Smooth muscle cells	Downregulates migration initiated by LTC_4_ (133).
		Fibroblasts	Downregulates production of MMP-3. IL-8. IL-6 induced by IL-1 beta, inhibits proliferation induced by CTGF (134, 135).
Resolvin E1	Eicosapentaenoic acid	Neutrophils	Downregulates generation of superoxide, trans-endothelial and epithelial migration (106, 136).
		Macrophages	Upregulates apoptotic neutrophil phagocytosis in a non-phlogistic manner (27).
		NK cells	Upregulates CMKLR1 receptor expression (127).
		Dendritic cells	Downregulates migration and production of IL-12 (34, 137).
Resolvin E3		Neutrophils	Downregulates infiltration (138).
Resolvin D1	Docosahexaenoic acid	Neutrophils	Downregulates transmigration (139).
		Macrophages	Downregulates TNF release as induced by LPS, upregulates apoptotic cell and allergen phagocytosis (140–142).
Protectin D1	Docosahexaenoic acid	Neutrophils	Downregulates IFN-gamma and TNF release, transmigration of PMNs, stimulates expression of CCR5 (9, 143, 144)
		Macrophages	Upregulates apoptotic PMN phagocytosis in a non-phlogistic manner (27).
Maresin 1	Docosahexaenoic acid	Bronchial epithelial cells	Downregulates cytokine production induced by dust (145).
		Regulatory T-cells	Upregulates T-reg cell formation and production of amphiregulin (146).
		ILC2	Downregulates production of IL-13 and upregulates production of amphiregulin (146).

CMKLR1, chemokine-like receptor 1; CCR5, chemokine receptor 5; CTGF, connective tissue growth factor; IL, interleukin; IFN gamma, interferon gamma; ILC2, innate lymphoid cell group 2; LTC_4_, leukotriene C_4_; LPS, lipopolysaccharide; NK, natural killer; PMN, polymorphonuclear leukocyte; MMP3, matrix metalloproteinase 3; PKC, protein kinase; TNF, tumor necrosis factor; SPM, specialized pro-resolving mediator; VEGF, vascular endothelial growth factor.

## Conclusion

In summary, it would appear that SPMs do not act alone via a purely immunological pathway but exert effects on dysbiosis as well. This assumes significant importance in terms of our understanding of periodontal disease, its etiopathology as well as treatment. It would be logical to argue that periodontal disease must be treated as an immunological imbalance created as a result of microbial insult and not just an isolated case of the latter infecting host tissues to cause the disease. This is evidenced quite clearly in the increased diversity and microbial load in periodontal disease when compared to exogenous infections. Clearly, there appears to be immunological factors at play which modify the bacterial diversity and numbers in periodontal disease. With such an understanding, it would be more appropriate to state that Periodontitis is an inflammatory disease initiated by bacteria.
